# Hematopoietic stem cell transplantation for autoimmune diseases in the time of COVID-19: EBMT guidelines and recommendations

**DOI:** 10.1038/s41409-021-01326-6

**Published:** 2021-05-24

**Authors:** Raffaella Greco, Tobias Alexander, Joachim Burman, Nicoletta Del Papa, Jeska de Vries-Bouwstra, Dominique Farge, Jörg Henes, Majid Kazmi, Kirill Kirgizov, Paolo A. Muraro, Elena Ricart, Montserrat Rovira, Riccardo Saccardi, Basil Sharrack, Emilian Snarski, Barbara Withers, Helen Jessop, Claudia Boglione, Ellen Kramer, Manuela Badoglio, Myriam Labopin, Kim Orchard, Selim Corbacioglu, Per Ljungman, Malgorzata Mikulska, Rafael De la Camara, John A. Snowden, On behalf of the European Society for Blood and Marrow Transplantation (EBMT) Autoimmune Diseases Working Party (ADWP), Infectious Diseases Working Party (IDWP), Pediatric Working Party (PWP), Joint Accreditation Committee of the International Society for Cellular Therapy (ISCT) and EBMT (JACIE), EBMT Nurses Group and Patient Advocacy Committee

**Affiliations:** 1 Unit of Hematology and Bone Marrow Transplantation, IRCCS San Raffaele Scientific Institute, Vita-Salute San Raffaele University, Milan, Italy; 2 Department of Rheumatology and Clinical Immunology, Charité - Universitätsmedizin Berlin, Corporate Member of Freie Universität Berlin, Humboldt-Universität zu Berlin, and Berlin Institute of Health, Berlin, Germany; 3 Department of Neuroscience, Uppsala University, Uppsala, Sweden; 4 Scleroderma Clinic, Dip. Reumatologia, ASST G. Pini-CTO, Milan, Italy; 5 Department of Rheumatology, Leiden University Medical Center, Leiden, The Netherlands; 6 Centre de Référence des Maladies Auto-Immunes Systémiques Rares d’Ile-de-France, Filière, Paris, France; 7 EA 3518, Université Denis Diderot, Paris, France; 8 Department of Internal Medicine, McGill University, Montreal, QC Canada; 9 Department for Internal Medicine II (Oncology, Hematology, Rheumatology and Immunology), University Hospital Tuebingen, Tübingen, Germany; 10 Kings Health Partners, Department of Haematology, Guys Hospital, London, UK; 11 N.N. Blokhin National Medical Center of Oncology, Institute of Pediatric Oncology and Hematology, Moscow, Russia; 12 Department of Brain Sciences, Imperial College London, London, UK; 13 Inflammatory Bowel Disease Unit, Gastroenterology Department, Hospital Clinic of Barcelona, Barcelona, Spain; 14 Institut d’Investigacions Biomèdiques August Pi i Sunyer (IDIBAPS), Centro de Investigación Biomédica en Red de Enfermedades Hepáticas y Digestivas (CIBERehd), Barcelona, Spain; 15 BMT Unit, Department of Haematology, Hospital Clinic, Institut d’Investigacions Biomèdiques August Pi i Sunyer (IDIBAPS); Institute Josep Carreras, Barcelona, Spain; 16 Department of Haematology, Careggi University Hospital, Florence, Italy; 17 Department of Neuroscience, Sheffield Teaching Hospitals NHS, Foundation Trust, Sheffield, UK; 18 NIHR Neurosciences Biomedical Research Centre, University of Sheffield, Sheffield, UK; 19 Department of Experimental and Clinical Physiology, Laboratory of Centre for Preclinical Research, Medical University of Warsaw, Warsaw, Poland; 20 LUX MED Oncology, Warsaw, Poland; 21 Polish Stem Cells Bank (PBKM), Warsaw, Poland; 22 Department of Haematology and Bone Marrow Transplant, Sydney, Australia; 23 Department of Haematology, Sheffield Teaching Hospitals NHS Foundation Trust, Sheffield, UK; 24 Patient Advocacy Committee, EBMT Executive Office, Eddific Dr. Frederic, Duran i Jorda, Barcelona, Spain; 25 EBMT Paris study office/CEREST-TC—Department of Haematology, Saint Antoine Hospital—INSERM UMR 938—Université Pierre et Marie Curie, Paris, France; 26 Department of Haematology, University Hospital Southampton and University of Southampton, Southampton, UK; 27 Department of Pediatric Hematology, Oncology and Stem Cell Transplantation, University of Regensburg, Regensburg, Germany; 28 Department of Cellular Therapy and Allogeneic Stem Cell Transplantation, Karolinska University Hospital Huddinge; Division of Hematology, Department of Medicine Huddinge Karolinska Institutet, Stockholm, Sweden; 29 Division of Infectious Diseases, University of Genoa (DISSAL) and Ospedale Policlinico San Martino, Genoa, Italy; 30 Department of Hematology, Hospital de la Princesa, Madrid, Spain; 31 Department of Oncology and Metabolism, University of Sheffield, Sheffield, UK

**Keywords:** Neurological disorders, Rheumatic diseases, Inflammatory bowel disease, Immunotherapy, Infectious diseases

## Abstract

Coronavirus disease-19 (COVID-19), caused by Severe Acute Respiratory Syndrome Coronavirus 2 (SARS-CoV-2), represents one of the biggest challenges of 21st century, threatening public health around the globe. Increasing age and presence of co-morbidities are reported risk factors for severe disease and mortality, along with autoimmune diseases (ADs) and immunosuppressive treatments such as haematopoietic stem cell transplantation (HSCT), which are also associated with adverse outcomes. We review the impact of the pandemic on specific groups of patients with neurological, rheumatological, and gastroenterological indications, along with the challenges delivering HSCT in adult and pediatric populations. Moving forward, we developed consensus-based guidelines and recommendations for best practice and quality of patient care in order to support clinicians, scientists, and their multidisciplinary teams, as well as patients and their carers. These guidelines aim to support national and international organizations related to autoimmune diseases and local clinical teams delivering HSCT. Areas of unmet need and future research questions are also highlighted. The waves of the COVID-19 pandemic are predicted to be followed by an “endemic” phase and therefore an ongoing risk within a “new normality”. These recommendations reflect currently available evidence, coupled with expert opinion, and will be revised according to necessary modifications in practice.

## Introduction

Since the onset of the COVID-19 pandemic [1], various reports detail its clinical manifestations and outcomes [2–6]. Delivery of HSCT requires maintenance of a complex infrastructure, quality assured according to a range of accreditation standards, and severely impacted during the pandemic. Under normal circumstances, autologous and allogeneic HSCT are performed in patients with severe ADs after careful balance of benefits and risks, with consideration of other non-transplant treatment options [7–10]. The majority of such patients have chronic diseases, which impact on quality of life and may shorten life expectancy. Transplant regimens used in autologous HSCT for ADs are generally more immunosuppressive than those used for other indications [8], and patients often receive immunomodulatory treatments prior and after HSCT. Immunocompromised patients are at elevated risk of complications from SARS-CoV-2 [11–17]. However, it remains uncertain whether conventional immunosuppressive treatments, glucocorticoid usage, and/or targeted-biologic disease modifying therapies (DMTs), are advantageous or detrimental [18].

As a basis for our guidelines and recommendations, we appraise the impact of the pandemic on patients with ADs, including non-HSCT treatments, along with the challenges delivering HSCT during the initial waves of the COVID-19 pandemic. The current phase of the COVID-19 pandemic is predicted to be followed by an “endemic” phase and therefore an ongoing risk within a “new normality”. There is therefore a need within the community for guidelines to restart HSCT programs, whilst maintaining quality and cautiously balancing risks and benefits against alternative treatment options in each AD. These guidelines and recommendations should be read in conjunction with general and AD-specific guidelines from European Society for Blood and Marrow Transplantation (EBMT) [19, 20]. They aim to provide useful information and general principles for national and international organizations and local clinical teams across relevant AD specialties whilst complementing guidelines and recommendations issued by other specialist societies.

## Methods

The recommendations (Tables 1–3) provided in this “living document” reflect currently available evidence, COVID-related guidelines regularly updated by EBMT [20], relevant AD specialists and HSCT societies, policies and procedures produced by national authorities as well as local and institutional policies, coupled with expert opinion from an international multidisciplinary team (MDT). Evidence was sourced from PubMed searches of original observations and key reviews, including the previous EBMT guidelines [7, 8, 19–21], and, where relevant, recent congress presentations.

**Table 1:** Summary of general recommendations [19, 20, 22, 25, 91].

Key factors	Recommendations	Remarks
Virus-related factors	• Surveillance of the local prevalence of virus in the community. • Continuous updating of the reproduction number “R” [26], reflecting the infectious potential of the disease. • Tracing of the national and regional alert status.	• In the event of “resurges” and peaks or local outbreaks. it is likely that HSCT for AD may again need to stop. • Patients and families need to be counseled about the possibility of short notice cancellation of their planned HSCT. • The key epidemiological parameters are the “R” rate, and the growth rate of the epidemic [26].
Hospital-related factors	• Availability of IPC and PPE, COVID-19 vaccination^a^ for the staff. • Testing and tracing of staff and patients (prior to mobilization and transplant). • Ability to create COVID secure facilities with clear pathways to separate patients from those that may have COVID. • Adequate supportive services for the HSCT program including ICU beds. • Suitable isolation facilities including single rooms with en suite facilities and for patients that tested positive for SARS-CoV-2 rooms with negative pressure or neutral pressure if this is not possible. • Backlog of patients with hematological malignancies who will take priority. • Discussing patients being considered for HSCT in appropriately constituted MDTs meeting; established treatment protocols for AD should be followed; mobilization with Cy provides additional disease control and requires consideration [7, 8]. • Visitors should generally not be admitted to transplant wards, except a single caregiver with negative swab for children.	• In many countries HSCT follows established pathway for adult elective care but patients may also need to access services urgently. • Where possible and clinically appropriate there should be separated care pathways for urgent and planned care, to eliminate the risk of nosocomial infection. Staff looking after COVID-19-positive patients should not be involved in face-to-face care of negative recipients. • Most hospitals have developed physically separate defined zones and cohorted staffing (reduced movement between COVID protected and non-protected areas). • All patients have to be screened at hospital entrance with questionnaire and temperature checks. • Patients should be tested for COVID-19 prior to starting the collection procedure, and before hospitalization for HSCT procedure, in order to protect the staff and other patients within the apheresis unit and the transplant unit from the nosocomial spread, and defer any transplant procedure in case of positivity for SARS-CoV-2 [20]. • It is important, however, that access to appropriate expertise is maintained and that pathways are also compliant with JACIE measures. • Risk minimization for other viral outbreaks is recommended, e.g., compliance of HCW with seasonal influenza vaccination. • Methods for communication between recipients, family members, and HCW such a video calls should be supported.
Patient-related factors	• Individual risk/benefit assessment and ability to give fully informed consent. • Ability to self-isolate, PPE compliance, home infrastructure to allow self-isolation, and agreement to comply with need to self-isolate. • Financial factors pertinent to the need to work from home for the first months following HSCT. • Ability to attend clinical appointments without using public transportation. • Post-transplant recovery and rehabilitation may be facilitated at home via telehealth [22, 92, 93], enabling early discharge from hospital if the patient can be safe and well-supported at home.	• Patients should be strongly advised to follow self-isolation and/or rigorous social distancing during and after HSCT. • The duration of this self-isolation should be carefully adapted on the COVID Alert Level^b^ within the community and the status of post-transplant immune reconstitution^c^, ranging from a minimum of 3 months after AHSCT (Alert Level 2), 6 months (Alert Level 3), 12 months (Alert Level 4), or until a full immune reconstitution (Alert Level 5). • Early influenza vaccination should be considered from 3 months after HSCT to decrease risk of hospitalization [7, 20, 86, 89]. Likewise, routine post-transplant anti-infective prophylaxis should be maintained as per guidance [7]. Moreover, optimizing vitamin D status plays an essential role in the immune system and may potentially have benefits in COVID-19 [94]. • Household contacts should receive COVID-19 vaccination^a^.
HSCT-related factors	• Consideration within the MDTs. • Potential recipients should self-quarantine at home for 14 days and be swabbed [20] (ideally within the 48 h and by molecular testing) prior to mobilization and transplant [20, 22]. • Potential recipients should not be transferred to the transplant ward and should not commence conditioning until a negative swab result has been reported. • In case when potential recipient tests positive for SARS-CoV-2, any transplant procedure (i.e., mobilization, collection, conditioning regimen) should be deferred [20]. In patients with mild or asymptomatic SARS-CoV-2 infection, deferral of at least 14 days after the first negative swab and symptoms clearance is required, with an additional negative swab before the start of conditioning. Similar minimum timeframes are advised from the time of last contact with a known COVID-positive contact. Deferral of HSCT for at least 3 months is recommended in patients with moderate–severe COVID-19.	• Although in a general HSCT context consideration of a modified mobilization procedure, such G-CSF alone, may be appropriate to avoid immunosuppression [22], in most AD protocols mobilization, the additional use of Cy provides additional disease control and may prevent disease flares. Cryopreservation is recommended. • Treatment in a clinical study, if available, should always be considered. • Immunocompromised patients can have a prolonged SARS-CoV-2 shedding (weeks or months), and recurrence of symptoms has been reported in a patient who became severely immunocompromised. • In the post-transplant period, patients with fever should be swabbed for SARS-CoV-2 as part of the workup for investigation of infection [7, 22, 91, 95]. All swabs should include testing for other respiratory viral pathogens [19]. Discussion of treatment for SARS-CoV-2 infection is beyond the scope of these guidelines but should follow the latest evidence available in local treatment guidelines and the responsibility of an expert in treating SARS-CoV-2 infection, yet with involvement of AD expert as the leading experts for the immunosuppressive therapy.

*AHSCT* autologous hematopoietic stem cell transplantation, *ADs* autoimmune diseases, *IPC* infection prevention and control, *PPE* personal protective equipment, *COVID* Coronavirus disease, *ICU* intensive care unit, *SARS-CoV-2* Severe Acute Respiratory Syndrome Coronavirus 2, *ATG* anti-thymocyte globulin, *GVHD* graft-versus-host disease, *HCW* healthcare workers, *Cy* cyclophosphamide, *MDTs* multidisciplinary teams.

^a^The protective role of active immunization might be limited in case of extensive circulation of viral strain with certain mutations.

^b^COVID-19 Alert Level [23, 24]—Level 1 (very low): COVID-19 is not known to be present; Level 2 (low): infection is present but the number of cases and transmission rate are low (R [26] below 1, growth rate [26] below 0 and average weekly number of new cases of <20 per 100 000 population); Level 3 (moderate): infection is epidemic in the general population but transmission is not high or rising exponentially (R [26] below 1, growth rate [26] below 0 and average weekly number of new cases of 20 or more per 100 000 population); Level 4 (high): infection is epidemic in the general population; transmission is high or rising exponentially (R [26] above 1 and growth rate [26] above 0); Level 5 (very high): as Level 4 but with material risk of healthcare services being overwhelmed.

^c^Post-transplant immune reconstitution [40, 96, 97] HSCT in ADs enables the regeneration of a new and non-disease-mediating immunity 40. The post-HSCT period is usually divided into the (1) pre-engraftment period (day 0 to days 15–45), (2) immediate post-engraftment period (engraftment to day +100), and (3) late post-engraftment period (days +100 to +365). In general, the neutrophil count recovers 2–3 weeks after HSCT. Recovery of B cells, natural killer (NK) cells, and CD8+ T cells is normally achieved in the first few weeks to 6 months, whereas CD4+ T-cell reconstitution is usually slower, where replenishment in adults may require up to 2 years post-HSCT [98, 99]. During the pre-engraftment period, the risk of opportunistic infection varies depending on the conditioning intensity (myeloablative or reduced intensity) [7, 40, 97]. In the post-engraftment period, the immune system is generally well-reconstituted and recovered in autologous HSCT recipients. Moreover, the degree of immune recovery (eg serum IgG concentrations> 4 g/L and CD4+ count >200 cells/μL) is associated with clinical outcomes, thus minimizing the risk of infection and healthcare attendance [40, 97, 100].

**Table 2:** Summary of recommendations for autologous HSCT in neurologic autoimmune diseases in the time of COVID-19.

	Strength of evidence and recommendations [7]	Clinical priority [22]	Maximum COVID-19 Alert Level [23, 24]	Minimum setting required [7]
Highly active relapsing remitting MS failing DMTs	S/I	1 2 3	4 3 2	Registry Prospective studies Prospective studies
Aggressive MS not previously treated with a full course of DMT	CO/II	1 2	3 2	Registry Registry
Progressive MS with active inflammatory component	CO/II	2	2	Registry
Progressive MS without active inflammatory component	GNR/III	N/A	N/A	N/A
Pediatric MS	CO/II	1 2	3 2	Registry Registry
Treatment-resistant CIDP	CO/II	2	2	Registry
Treatment-resistant NMOSD	CO/II	1 2	3 2	Registry Registry
Treatment-resistant SPSD	CO/II	1 2	3 2	Registry Registry
Rare IMNDs and treatment-resistant systemic ADs	CO/II	2	2	Registry

Strength of evidence of clinical efficacy [7]—Grade I: evidence from at least one well-executed randomized trial, Grade II: evidence from at least one well-designed clinical trial without randomization; cohort or case-controlled analytic studies, Grade III: evidence from opinions of respected authorities based on clinical experience. Recommendations^7^: *S* standard of care, *CO* clinical option, *GNR* generally not recommended.

Clinical priority (as determined by a relevant multidisciplinary team) [22]—1: high, delaying the HSCT procedure presents a high risk of disease progression, morbidity or mortality, 2: intermediate, there is a risk of disease progression or clinical complications if HSCT is delayed significantly, 3: low, the risk of disease progression or clinical complications if HSCT is significantly delayed is low.

COVID-19 Alert Level [23, 24]—Level 1 (very low): COVID-19 is not known to be present; Level 2 (low): infection is present but the number of cases and transmission rate are low (R [26] below 1, growth rate [26] below 0 and average weekly number of new cases of less than 20 per 100 000 population); Level 3 (moderate): infection is epidemic in the general population but transmission is not high or rising exponentially (R [26] below 1, growth rate [26] below 0 and average weekly number of new cases of 20 or more per 100 000 population); Level 4 (high): infection is epidemic in the general population; transmission is high or rising exponentially (R [26] above 1 and growth rate [26] above 0); Level 5 (very high): as Level 4 but with material risk of healthcare services being overwhelmed.

Setting [7]—prospective studies: randomized controlled trials (RCTs) and other clinical trials, including RAM-MS, STAR-MS, NET-MS, COAST, BEAT-MS, or prospective non-interventional studies (NIS) including OMST [6]. Registry: reporting data to EBMT registry (or equivalent international registry, e.g., CIBMTR).

*HSCT* autologous hematopoietic stem cell transplantation, *MS* multiple sclerosis, *DMT* disease modifying therapies, *CIDP* chronic inflammatory demyelinating polyradiculoneuropathy, *NMOSD* neuromyelitis optica spectrum disorder, *SPSD* stiff person spectrum disorder, *IMNDs* immune-mediated neurological disorders, *ADs* autoimmune diseases, *CIBMTR* Center for International Blood and Marrow Transplant Research, *N/A* not applicable.

**Table 3:** Summary of recommendations for autologous HSCT in RMDs in the time of COVID-19.

	Strength of evidence and recommendations [21]	Clinical priority [22]	Maximum COVID-19 Alert Level [23, 24]	Minimum setting required [8]
SSc	S/I	1 2	4 3	Prospective studies Registry
SLE	CO/II	1 2	3 2	Registry
Vasculitis	CO/II	1 2	3 2	Registry
Polymyositis– dermatomyositis	CO/II	1 2	3 2	Registry
RA or JIA	CO/II	1 2	3 2	Registry

Strength of evidence of clinical efficacy [21]—Grade I: evidence from at least one well-executed randomised trial, Grade II: evidence from at least one well-designed clinical trial without randomization; cohort or case-controlled analytic studies, Grade III: evidence from opinions of respected authorities based on clinical experience. Recommendations [21]: *S* standard of care, *CO* clinical option, *GNR* generally not recommended.

Clinical priority (as determined by a relevant multidisciplinary team) [22]—(1) high, delaying the HSCT procedure presents a high risk of disease progression, morbidity, or mortality, (2) intermediate, there is risk of disease progression or clinical complications if HSCT is delayed significantly, (3) low, the risk of disease progression or clinical complications if HSCT is significantly delayed is low.

COVID-19 Alert Level [23, 24]—Level 1 (very low): COVID-19 is not known to be present; Level 2 (low): infection is present but the number of cases and transmission rate are low (R [26] below 1, growth rate [26] below 0 and average weekly number of new cases of <20 per 100 000 population); Level 3 (moderate): infection is epidemic in the general population but transmission is not high or rising exponentially (R [26] below 1, growth rate [26] below 0 and average weekly number of new cases of 20 or more per 100,000 population); Level 4 (high): infection is epidemic in the general population; transmission is high or rising exponentially (R [26] above 1 and growth rate [26] above 0); Level 5 (very high): as Level 4 but with material risk of healthcare services being overwhelmed.

Setting—prospective studies: randomized controlled trials (RCTs) and other clinical trials, or prospective non-interventional studies (NIS). Registry: reporting data to EBMT registry (or equivalent international registry, e.g., CIBMTR).

*HSCT* autologous hematopoietic stem cell transplantation, *RMDs* rheumatic and musculoskeletal diseases, *SSc* systemic sclerosis, *SLE* systemic lupus erythematosus, *RA* rheumatoid arthritis, *JIA* juvenile idiopathic arthritis, CIBMTR Center for International Blood and Marrow Transplant Research, *N/A* not applicable.

As per other EBMT guidelines and recommendations, level of evidence for the efficacy of autologous HSCT in ADs is systematically classified in three categories of recommendations where HSCT should be considered (S/CO/GNR—see Tables 2 and 3, and related footnotes) [7, 21]. Strength of evidence supporting the assignment of a particular category of recommendations is graded (levels I, II, and III) based on consideration of health benefits, side effects, and risks and balanced against the non-HSCT options. Clinical priority, as determined by a relevant MDT [22], has been classified as high, intermediate, or low, while the COVID-19 Alert Level [23, 24] as very low, low, moderate, high, or very high (see Tables 2 and 3, and related footnotes).

### Preliminary analysis of the impact of COVID-19 outbreak on HSCT programs for ADs

**Fig. 1:**
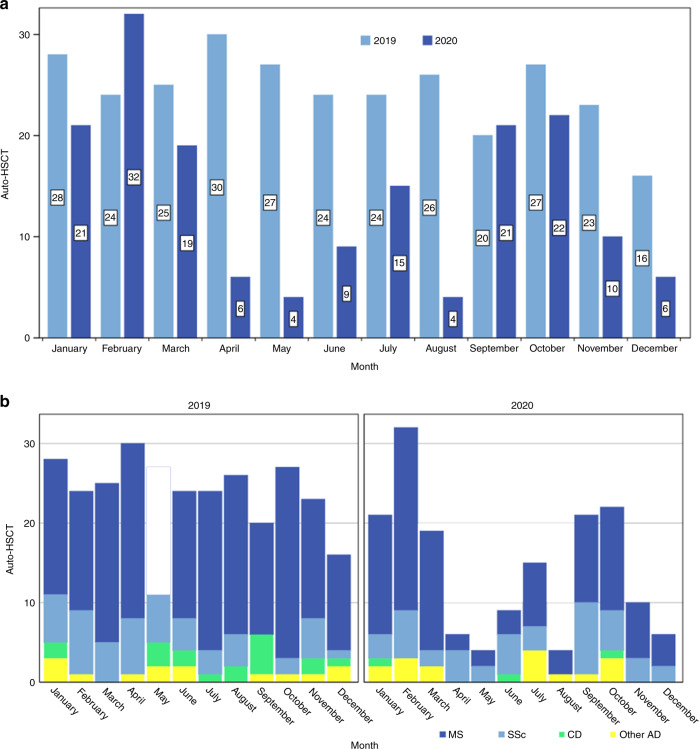
Autologous hematopoietic stem cell transplant (HSCT) for autoimmune diseases (ADs) 2019–2020, reflecting the impact of the COVID-19 pandemic in Europe. **a** Numbers of autologous HSCT in January to December 2020 are compared with the numbers in January to December 2019; **b** 2019–2020 transplants are also represented according to disease indication (MS: deep blue; SSc: light blue; CD: green; other ADs: yellow). auto-HSCT autologous hematopoietic stem cell transplantation, AD autoimmune disease, MS multiple sclerosis, SSc systemic sclerosis, CD Crohn’s disease.

Across the EBMT registry, we investigated the impact of the COVID-19 outbreak on transplant activity for ADs. When the transplant activity for ADs during the pandemic was compared with the corresponding time in 2019, a total of 116 patients received an autologous HSCT between March and December 2020, while 242 patients received autologous HSCT the previous year in the same time frame (Fig. 1). Compared to 2019, transplant activity for ADs decreased by 52%. Nevertheless, indications remained unchanged with multiple sclerosis (MS) and systemic sclerosis (SSc) comprising 80% of transplants in ADs reported to the EBMT in 2020, being HSCT an integral and standard-of-care part of their treatment algorithms [21].

### General recommendations for restarting HSCT program in ADs

All AD patients considered for HSCT should be carefully discussed at the local MDT meeting, with input from HSCT and AD specialists and consideration of alternative treatments [20]. If HSCT treatment is in the best interests of patients, established treatment protocols for AD should be followed, as per guidelines and evidence-based appraisals.

The ongoing learning curve will need to be extended into longer-term modifications in clinical practice, with the “restoration and recovery” or “reset” periods, during which SARS-CoV-2 minimization can be completed, aiming to overcome any further “resurges” and peaks. Consequently, HSCT programs must be ready to rapidly adapt to this change following the course of the pandemic and be able to prioritize the process of delivering HSCT according to clinical urgency [25], depending on virus, hospital, patient, and HSCT-related factors (Table 1) [22, 25].

Nurses have a key role in explaining all the related precautions, including strict adherence to local policies relating to visiting. Clear written information for patients and caregivers should be provided, including measures to minimize the risk of infection, and need for a dedicated caregiver, having low risk of SARS-CoV-2 exposure, in the first months after HSCT. Patients intending to travel abroad for HSCT should consider carefully what arrangements are in place to minimize the risk but also the provision of care post discharge, especially if there are any potential travel restrictions between the country of treatment and their home.

Since the COVID-19 situation varies substantially between and within countries, we recognize that centers are mandated to follow guidelines, policies, and procedures decided by national authorities as well as local and institutional policies. Special consideration should be made when the home base or country of the patient is different from the HSCT center, where R numbers [26] and clinical practice, including in relation to precautions, may differ. As such arrangements will be individualized, there is a recommendation that patients are systematically discussed by and between MDTs at both sites, and, after the HSCT procedure, there is a clear communication between the transplant center and the receiving site and their clinicians for ongoing follow up and advice.

### Considerations and recommendations for HSCT in neurologic autoimmune diseases

Immune-mediated neurological disorders (IMNDs) affect the central and the peripheral nervous systems resulting in a range of diseases including MS, neuromyelitis optica spectrum disorder (NMOSD), stiff person spectrum disorder, myasthenia gravis, chronic inflammatory demyelinating polyradiculoneuropathy (CIDP), autoimmune encephalopathies, and others [27].

The limited available evidence suggests that IMNDs and their treatments affect the susceptibility to or the severity of COVID-19. Patients with MS have increased risk of several types of infections compared to the general population particularly if they are on B-cell depleting therapies, such as rituximab [28, 29]. An earlier study from Italy reported on 232 MS patients from 38 centers with confirmed or suspected COVID-19 [30], did not show any significant association between previous DMT exposure and COVID-19 severity. Of those patients, 223 had mild, 4 had severe, and 6 had critical infection.

In a registry-based study of 347 patients with MS, age, EDSS score, and obesity were found to be independent risk factors for severe COVID-19, although no association was found between DMTs exposure and COVID-19 severity [31]. Another study suggested that the incidence of COVID-19 in MS patients was not more than that of the general population, but the risk of hospitalization in these patients was higher than estimated for the disease [32]. The prevalence and impact of COVID-19 across Europe were assessed in the 399 patients with MS taking part in the RADAR-CNS programme [33]; of those 21.8% reported major symptoms suggestive of COVID-19, mainly associated to alemtuzumab and cladribine treatments. In the MS Global Data Sharing Initiative [34], clinician-reported data from 21 countries on 1540 patients in which 776 (50.4%) had confirmed COVID-19, and confirmed that older age, progressive MS, and higher EDSS scores were associated with higher frequencies of severe outcomes. Anti-CD20 DMTs, ocrelizumab, and rituximab were positively associated with hospital and ICU admission and the need for artificial ventilation compared to all other DMTs.

Therefore, patients with MS seem to be affected by the same risk factors as the general population, high EDSS scores appear to be an MS-specific risk factor and the majority of DMT treatment does not seem to be associated with a particularly poor prognosis.

Data related to other IMNDs are limited. CARE-MG registry reported worsening of myasthenic control requiring rescue therapy in the setting of COVID-19 in 36 of 91 patients. Complete recovery or discharge to home was reported in 39 (43%) patients whereas 22 (24%) patients died due to COVID-19 [35]. The clinical course and outcome of patients with COVID‐19 and NMOSD seem to be variable [36–38] and also exacerbations of CIDP have been reported [39].

The use of autologous HSCT in the treatment of IMNDs is expanding and becoming increasingly evidence-based [7, 40–42]. Recently, the first phase III randomized controlled trial [41] in MS has demonstrated its efficacy in patients with active relapsing remitting disease failing standard DMTs. A number of phase II non-randomized trials have also shown its safety and efficacy in treatment-resistant CIDP [43], SPS [44], and NMOSD [45], whilst definitive data are less readily available [7, 27, 42, 46]. The conditioning regimens usually employed in neurological ADs range from high-dose cyclophosphamide and the combination of BCNU, Etoposide, ARA-C and Melphalan, both associated with serotherapy with either anti-thymocyte globulin (ATG), rabbit or horse, or monoclonal antibodies targeted to lymphocyte subsets, such as alemtuzumab or rituximab [7]. The different intensity of the regimens results in corresponding degrees of immunosuppression. In any case, with any type of regimen, a patient would be considered at high risk in case of SARS-CoV-2 infection [39, 47, 48].

The most appropriate choice of conditioning regimen should be addressed for each individual patient, after a careful assessment of diagnosis, disease stage, and overall clinical condition at baseline. Therefore the use of this treatment should be restricted to patients with a clear risk/benefit ratio based on treatment-resistant disease entity, according to the level of evidence for autologous HSCT efficacy in this particular disease, defined clinical priorities [22], and local COVID-19 Alert Levels (Table 2) [23].

All treatment-related decisions should be made by MDTs and patients should be treated within clinical trials if available or as part of well-defined registry studies to allow longitudinal collection of efficacy and safety data of the various treatment options. Patients with severe co-morbidities known to be associated with poor COVID-19 outcomes should not be considered for HSCT during the pandemic.

### Considerations and recommendations for HSCT in rheumatic diseases

The COVID-19 pandemic has considerable impact on different aspects of the management of patients with rheumatic and musculoskeletal diseases (RMDs). Chronic suppression of immune functions, both with synthetic or biologic DMTs, is the cornerstone of treatment in those indications [49–53]. Alongside, autologous HSCT has emerged as an established treatment option for some indications where effective drug therapy is not available, even in the biological era. Particularly, three randomized controlled trials (ASSIST, ASTIS and SCOT) have demonstrated the superiority of HSCT compared to monthly IV bolus of cyclophosphamide in patients with early rapidly progressive SSc in terms of event-free and overall survival, improvement of skin fibrosis, and evidence for benefits on pulmonary function [54–56]. SSc is now considered a standard indication for autologous HSCT [21, 57]. Other diseases in which the use of HSCT is supported by evidence from non-randomized controlled trials and is regarded as treatment option include systemic lupus erythematosus (SLE), vasculitides, polymyositis/dermatomyositis, and both rheumatoid and juvenile idiopathic arthritis [21].

Chronic use of DMTs may be associated with an increased risk of infection-related morbidity and mortality in RMDs [58, 59]. However, despite this notion, accumulating data from larger case series, national and international registries suggest that patients with RMDs neither have an increased risk of developing SARS-CoV-2 infection nor do they have a worse prognosis compared to the general population [60–63]. Only RMD patients on high-dose corticosteroids, but not methotrexate and biologic DMTs, non-steroidal anti-inflammatory drugs and antimalarial drugs had a higher risk of SARS-CoV-2 infection or hospitalization [60–62, 64, 65]. However, recent data from the French RMD COVID-19 cohort including 694 adults indicated for the first time an increased risk for severe infection in patients receiving mycophenolate mofetil or rituximab [65]. Conversely, RMD patients on a background tumor necrosis factor (TNF) inhibitor had an adjusted 60% reduction in risk of hospitalization [62], and recently the FDA authorized the emergency use of baricitinib in certain hospitalized patients with COVID-19. Overall, risk factors for more severe COVID-19 in RMD patients include older age and comorbid conditions [65]. The only rheumatic disease diagnosis with odds of hospitalization significantly different from other RMDs seems to be SLE. Data from the Global Rheumatology Alliance (GRA) Registry indicated that lupus patients were at 80% increased risk of hospitalization [62]. Patients with SSc are largely underrepresented in international COVID-19 registries and their risk during COVID-19 pandemic is evaluated incompletely. Nevertheless, first single-center studies and personal observations indicated that incidence of confirmed SARS-CoV-2 infections was low and severe complications, including death, were rare. Only one death and 11 infections related to COVID-19 have been reported among 526 Italian SSc patients [66]. Overall, 390 (10.5%) died out of 3729 RMD patients included in the GRA physician COVID-19 registry [67]. Provisional recommendations for the management of RMD patients were published based on expert consensus from international task forces of the EULAR [68] and ACR [69]. Both recommendations strongly suggest continuing the DMTs treatment in RMD patients who do not have suspected or confirmed COVID-19. In addition, management of RMD patients following SARS-CoV-2 exposure and documented infection, and the use of immunosuppressive drugs, should be multidisciplinary, discouraging the off-label use of DMTs outside the context of clinical trials [68].

The use of HSCT in RMDs must be carefully weighed against the risk of the procedure, should be based on the strengths of evidence per indication and ideally performed as part of a clinical study. Particularly, access to intensive care medicine and resources for appropriate screening procedures, including the recommended cardiopulmonary screening assessments for SSc patients should be available [63]. Appropriate candidates for autologous HSCT during COVID-19 pandemic should therefore be those with high clinical priority, reflected by life-threatening and otherwise refractory courses of the disease, i.e., unresponsiveness or lack of tolerability to standard or alternative DMTs therapies, in which delaying the HSCT procedure may be associated with a high risk of disease progression, morbidity, or mortality (Table 3).

In summary, continuation with autologous HSCT for RMD patients during COVID-19 pandemic can be recommended, presupposing a risk adjustment according to local SARS-CoV-2 infection rates and medical resources, strengths of evidence of HSCT per indication, lack of alternative therapies and adoption of a prioritization process, delivering HSCT based on clinical urgency.

### Considerations and recommendations for gastrointestinal diseases

In gastroenterology, the main area application of autologous HSCT has been in inflammatory bowel diseases (IBDs), particularly Crohn’s disease (CD) [9, 10]. Although the COVID-19 pandemic has led to substantial concerns for patients with IBDs, as a high proportion of them receive immunosuppressive therapies [70], similar COVID-19 rates and no increased mortality have been reported as compared to the general population [71–73]. Corticosteroids may pose significant risk to IBD patients with COVID-19 [72]. In addition, recent results demonstrated that thiopurine treatment, either as monotherapy or in combination with TNF inhibitors, was associated with increased risk of severe COVID-19 [74, 75]. Anti-TNF therapy, anti-integrins, and anti-IL12/23 have not been associated with increased mortality [71, 73].

Recommendations in the management of IBD suggest that medical treatments should be re-evaluated in SARS-CoV-2-positive patients and corticosteroid therapy should be re-evaluated regardless of symptoms. A goal should be to treat active disease and maintain remission, while adopting the same protective measures as the general population. In addition, nonurgent surgeries and endoscopic procedures should be postponed [76]. The use of autologous HSCT should be restricted to patients with a clear risk/benefit ratio, according to strengths of evidence of HSCT per indication, clinical priorities [22], and local COVID-19 Alert Levels (Table 4) [23].

**Table 4:** Summary of recommendations for autologous HSCT in IBDs in the time of COVID-19.

	Strength of evidence and recommendations [21]	Clinical priority [22]	Maximum COVID-19 Alert level [23, 24]	Minimum setting required [8]
Crohn’s disease	CO/II	1 2	3 2	Registry
RCD II	CO/II	1 2	3 2	Registry

Strength of evidence of clinical efficacy [21]—Grade I: evidence from at least one well-executed randomised trial, Grade II: evidence from at least one well-designed clinical trial without randomization; cohort or case-controlled analytic studies, Grade III: evidence from opinions of respected authorities based on clinical experience. Recommendations [21]: *S* standard of care, *CO* Clinical option, *GNR* generally not recommended.

Clinical priority (as determined by a relevant multidisciplinary team) [22]— (1) high, delaying the HSCT procedure presents a high risk of disease progression, morbidity or mortality, (2) intermediate, there is risk of disease progression or clinical complications if HSCT is delayed significantly, (3) low, the risk of disease progression or clinical complications if HSCT is significantly delayed is low.

COVID-19 Alert Level [23, 24]—Level 1 (very low): COVID-19 is not known to be present; Level 2 (low): infection is present but the number of cases and transmission rate are low (R [26] below 1, growth rate [26] below 0 and average weekly number of new cases of <20 per 100,000 population); Level 3 (moderate): infection is epidemic in the general population but transmission is not high or rising exponentially (R [26] below 1, growth rate [26] below 0 and average weekly number of new cases of 20 or more per 100,000 population); Level 4 (high): infection is epidemic in the general population; transmission is high or rising exponentially (R [26] above 1 and growth rate [26] above 0); Level 5 (very high): as Level 4 but with material risk of healthcare services being overwhelmed.

Setting—prospective studies: randomized controlled trials (RCTs) and other clinical trials, or prospective non-interventional studies (NIS). Registry: reporting data to EBMT registry (or equivalent international registry, e.g., CIBMTR).

*HSCT* autologous hematopoietic stem cell transplantation, *IBD* inflammatory bowel diseases, *RCD* refractory celiac disease, *CIBMTR* Center for International Blood and Marrow Transplant Research, *N/A* not applicable.

### Considerations and recommendations for pediatric population

Only limited data are available on risks of HSCTs in pediatric ADs [76, 77] during the COVID-19 outbreak [30]. There was no significant association between previous DMTs and COVID-19 severity in children [77]. Therefore, children post-HSCT for ADs usually have a story of intensive immunosuppression and are at potential risk during the COVID-19 outbreak [13]. A rapid global response for children with cancer was published [78]. Feasibility of HSCT for pediatric ADs during the COVID-19 outbreak was demonstrated, provided there is the availability of hospital resources. Therefore, during the pandemic outbreak, the indication for HSCT in children with ADs must be restricted to patients with a clear risk/benefit ratio [78]. In children, it could be severe “malignant” forms of neurological ADs, severe progressive systemic diseases, CD, and immune cytopenias.

Considering the better outcomes of HSCT in children during the COVID-19 outbreak, a less restrictive approach could be applied [79]. As patients with pediatric ADs are mostly transplanted in the same departments as patients with malignancies, priority must be given to these children followed by patients with severe ADs [78]. Centers must be very careful using immunotherapy such as ATG as well as monoclonal antibodies [80]. Preferably, only one parent or guardian, identified as the primary caregiver, and with a recent negative swab for SARS-CoV-2, may be present in the room with a pediatric patient, requesting a visitor exception to the local care team.

### Special recommendations for vaccinations

The response to the pandemic includes efforts to develop safe and effective vaccines [81] with an unprecedented urgency and large-scale investment of human and financial resources [82].

All the main arms of the immune response are involved in the generation of protective immunity to SARS-CoV-2: innate immune responses as well as adaptive immunity, including humoral and T-cell responses. SARS-CoV-2 has the ability to suppress innate immune responses [81]. Antibody responses to the Coronavirus spike (S) protein develop during COVID-19 infection in the majority of subjects [83]. Emerging evidence indicates that T-cell mediated immunity may be critically important against COVID-19 [81, 84, 85]. Collectively, these data suggest that individuals with compromised innate or adaptive immunity may not only be at risk of more severe COVID-19 disease but also at higher risk of lacking protective immunity from infection or vaccination.

The guidelines of the 2017 European Conference on Infections in Leukaemia provide general recommendations for vaccination in HSCT recipients [86]. Recently, EBMT has provided specific COVID-19 vaccine recommendations [20]. The vaccines against COVID-19 currently approved for use in EU, following review by EMA, can be grouped in two platforms: recombinant replication-defective viral-vectored vaccines and mRNA-based vaccines [20, 81]. No evidence is published to inform us on the safety and efficacy of any COVID-19 vaccine in HSCT recipients. Based on inferences from other current evidence [20, 87, 88], we suggest that live-attenuated COVID-19 vaccines or vectored by live-attenuated viruses should be contraindicated in HSCT recipients. For all other COVID-19 vaccine types, we suggest that vaccination could be considered from 3 months after HSCT. Follow-up after vaccination is therefore important. Continued precautions should be taken based on the pandemic situation in the society [20, 89]. Individuals vaccinated before HSCT will almost certainly lose protective immunity after HSCT and may require revaccination. Clinical trials in HSCT patients are needed to establish the safety and immune response of vaccines against COVID-19.

### Data reporting

Routine data reporting should continue into the EBMT registry, and, alongside, we strongly encourage center participation in the ongoing EBMT-wide prospective survey on the impact of COVID-19 in HSCT recipients [19].

### Clinical trials

With respect to clinical trials, special consideration should be made by trial management groups, oversight committees, sponsors, and local principal investigators in the best interests of patients in relation to the trial protocol and recruitment during the pandemic [90]. Even so, clinical trials provide a means of actively monitoring the impact of the COVID-19 pandemic on outcomes.

### Quality and accreditation

In EBMT, quality in HSCT and cellular therapy is assured through JACIE accreditation, which has been central to EBMT recommendations for HSCT in ADs [8]. Elsewhere, equivalent quality assurance is provided by FACT, based on harmonized FACT—JACIE standards. Therefore, we recommend that HSCT procedure should only be carried out in experienced centers with an active accreditation by JACIE or FACT [7].

As the situation may change over time, the current statements should be reviewed at regular intervals and read in conjunction with general EBMT guidelines [20] on the COVID-19 pandemic, which are continually updated [20], along with local and national guidance.

## Conclusions

Although current evidence suggests that HSCT is a valid treatment option in the management of selected patients with ADs, its intense immunosuppression could expose to increased risks during the current COVID-19 pandemic [13, 47, 48]. Therefore the use of autologous HSCT during the pandemic should be restricted to AD patients with a clear risk/benefit ratio based on disease entity, level of evidence for HSCT efficacy in this setting, defined clinical priorities [22], and local COVID-19 Alert Levels (Tables 2–4) [23]. All patients being considered for HSCT should be discussed in appropriately constituted MDT meetings (including transplant and disease specialists) with individual assessment of risks and benefits of HSCT as best as possible and considering the current and predicted geographical variations in the pandemic related to transplant center, referral center, and home locality of the patient (which may be different). HSCT programs must be ready to rapidly adapt to change following the course of the pandemic. Updates of this COVID-19-specific guidance will be incorporated within updates to recommendations from EBMT for specific ADs.
